# Metabolomic Profiling in *LRRK2*-Related Parkinson's Disease

**DOI:** 10.1371/journal.pone.0007551

**Published:** 2009-10-22

**Authors:** Krisztina K. Johansen, Lei Wang, Jan O. Aasly, Linda R. White, Wayne R. Matson, Claire Henchcliffe, M. Flint Beal, Mikhail Bogdanov

**Affiliations:** 1 Department of Neuroscience, Norwegian University of Science and Technology, Trondheim, Norway; 2 Department of Neurology, St. Olav’s Hospital, Trondheim, Norway; 3 Bedford VAMC, Bedford, Massachusetts, United States of America; 4 Department of Neurology and Neuroscience, Weill Medical College of Cornell University, New York, New York, United States of America; National Institutes of Health, United States of America

## Abstract

**Background:**

Mutations in *LRRK2* gene represent the most common known genetic cause of Parkinson's disease (PD).

**Methodology/Principal Findings:**

We used metabolomic profiling to identify biomarkers that are associated with idiopathic and *LRRK2* PD. We compared plasma metabolomic profiles of patients with PD due to the *G2019S LRRK2* mutation, to asymptomatic family members of these patients either with or without *G2019S LRRK2* mutations, and to patients with idiopathic PD, as well as non-related control subjects. We found that metabolomic profiles of both idiopathic PD and *LRRK2* PD subjects were clearly separated from controls. *LRRK2* PD patients had metabolomic profiles distinguishable from those with idiopathic PD, and the profiles could predict whether the PD was secondary to *LRRK2* mutations or idiopathic. Metabolomic profiles of *LRRK2* PD patients were well separated from their family members, but there was a slight overlap between family members with and without *LRRK2* mutations. Both *LRRK2* and idiopathic PD patients showed significantly reduced uric acid levels. We also found a significant decrease in levels of hypoxanthine and in the ratios of major metabolites of the purine pathway in plasma of PD patients.

**Conclusions/Significance:**

These findings show that *LRRK2* patients with the *G2019S* mutation have unique metabolomic profiles that distinguish them from patients with idiopathic PD. Furthermore, asymptomatic *LRRK2* carriers can be separated from gene negative family members, which raises the possibility that metabolomic profiles could be useful in predicting which *LRRK2* carriers will eventually develop PD. The results also suggest that there are aberrations in the purine pathway in PD which may occur upstream from uric acid.

## Introduction

Parkinson's disease (PD) was long considered to be largely idiopathic, but several genetic factors have been reported during the last decade. *LRRK2, Parkin* and *PINK1* genes have the most clinical relevance because of their comparatively high frequency [1]–[4]. The *6055G >A* mutation in the *LRRK2* gene, resulting in a *G2019S* substitution in the LRRK2 protein is common, though the frequency varies dependent on ethnic group [5]–[7]. In central Norway, about 3% of all PD cases have been found to carry this mutation, although the penetrance in affected families is incomplete [8].

The discovery of PD genes has greatly improved our understanding of PD and could possibly provide new strategies for treatment. The diagnosis of PD is still mainly based on clinical features, and a definitive diagnosis can only be confirmed by autopsy findings. Disease progression can be followed with functional neuroimaging techniques such as PET and SPECT [9], [10], but these techniques have limited availability, are expensive, and are not used in daily clinical practice.

Several biochemical markers have been assessed as potential biomarkers, such as dopamine metabolites in the CSF of PD patients, but do not correlate with PD severity [11]. Decreased levels of α-synuclein in CSF, and an elevated level of the oligomeric protein in plasma have been reported [12], [13], but neither is recognized as a diagnostic marker. Several studies are currently in progress to find biomarkers using genomic, proteomic or metabolomic approaches [14], [15].

Metabolomics is the comprehensive analysis of low molecular weight molecules within a particular biological sample, followed by organization for data mining and bioinformatics [16], and has been used to study several illnesses, including heart disease [17], type 2 diabetes [18], cancer [19], and nervous system diseases such as schizophrenia [20], amyotrophic lateral sclerosis [21], and Huntington's disease [22]. We recently found that idiopathic PD patients have metabolomic phenotypes which differ from control subjects [14].

Clinically, *LRRK2* PD patients are indistinguishable from idiopathic PD patients [8], [23], [24]. Genotyping can differentiate between *LRRK2* and idiopathic PD patients after onset of clinical symptoms. However, it provides incomplete information about possible development of PD in asymptomatic gene carriers, due to incomplete penetrance. Therefore, it is important to find biomarkers in mutation carriers which may predict the development of the clinical symptoms.

In the present study we used a metabolomics approach to define plasma metabolomes associated with *LRRK2* PD, idiopathic PD, asymptomatic *LRRK2 G2019S* carriers, and normal control subjects. Both untargeted and targeted (including 34 major known metabolites of the purines, tyrosine, and tryptophan pathways) approaches were performed to thoroughly investigate the differences between *LRRK2* and idiopathic PD, as well as between controls and all PD patients.

## Methods

### Patients

A total of 99 subjects were enrolled in this study: PD patients without any known mutations (idiopathic, n = 41) and PD patients carrying the *G2019S* mutation in *LRRK2* gene (n = 12). The healthy family members from the mutation carrier PD cases were invited to participate in this study and 21 of them tested positive for the mutation (mut+) and 10 of them were negative (mut−). Additionally, 15 control subjects who were healthy with no signs of any movement disorder, and were not in the family or a spouse of the *LRRK2 G2019S* cases, were recruited from the same area. Table 1 presents the demographic data. All the PD patients were examined and followed up by one neurologist (J.O.A.). Clinical criteria for diagnosis required the presence of at least two cardinal motor signs: asymmetric resting tremor, bradykinesia and rigidity, as well as a good response to levodopa and absence of other atypical features and causes of parkinsonism [25]. A complete neurological examination was performed on the healthy family members without any clinical signs of parkinsonism.

**Table 1:** The clinical data of the enrolled subjects.

	N	Age	Sex (M/F)	AAO	Duration
**Idiopathic PD**	41	64.8 (45–77)	25/16	53.4 (30–71)	11.2 (2–33)
***LRRK2*** ** PD**	12	72.8 (53–88)	5/7	61.1 (43–75)	11.7 (2–27)
**Mut**+	21	55.7 (28–81)	15/6	-	-
**Mut**−	10	54.6 (41–78)	7/3	-	-
**Control**	15	66.4 (54–88)	8/7	-	-

Idiopathic PD: patients without any known mutations; *LRRK2* PD: patients carrying the *G2019S* mutation in *LRRK2*; Mut+: healthy family members with *G2019S* mutation in *LRRK2*; Mut−: healthy family members without mutation; Controls: healthy subjects without any sign of neurological diseases; AAO: age at disease onset.

All the patients included in this study were tested for known PD-related mutations and none of the patients with idiopathic PD were found to have any such mutations. The 12 *LRRK2* PD patients were heterozygous carriers for the *G2019S* mutation, from 9 unrelated families. The treatment of the two PD groups consisted for the most part of a combination of levodopa and dopamine agonists, but 8 idiopathic patients used selegiline, and 3 used antipsychotic medicines. No patients with the mutation were treated with the latter two types of medication. Table 2 shows a list of the medications used by the patients. The family members of *LRRK2* PD patients were screened specifically for the *G2019S* mutation, as were the healthy control subjects. The mutation was not found in any of the controls, but was found in twenty one family members.

**Table 2:** The medication of PD patients.

	Idiopathic PD		*LRRK2* PD		
	N	Dosage (mg)	N	Dosage (mg)	*P* value
Levodopa monotherapy	13	521±387	8	337±74	0.151
Dopamine agonist monotherapy, LEDD.	3	300±0	0		
Combination of levodopa and dopamine agonist, LEDD	24	842±326	4	1072±687	0.869
Total dose, LEDD.	40	697±380	12	583±513	0.104

Dosages of dopamine agonist are calculated in levodopa equivalent dosages, LEDD. One *de novo* idiopathic patient was without any medication; total number of idiopathic patient is 41.

### Ethics statement

Written informed consent was acquired from all subjects participating in this study, according to the declaration of Helsinki. The study was approved by the Regional Committee for Medical Research Ethics, Central Norway. The protocol for analysis of the samples at the Bedford VAMC was approved by the Bedford VAMC IRB.

### Sample preparation and analysis

Plasma samples were prepared for analysis by extraction in acidified acetonitrile and analyzed by LCECA as previously described [14], [21], [26]–[28]. During the sample preparation, pools were created from equal volumes of subaliquots of all samples in the study. Pools and duplicates were used to assess the precision of the entire data set and to control the overall performance of the analytical method. The pools also served as references for time normalization (peak stretching) and were used to express the concentrations of each peak in the samples as a percentage of the concentration of those peaks in the averaged pool. The replicate analyses of the pool also provided an estimate of the coefficient of variation associated with each peak, that is, the standard deviation of the peak height across pool replicates normalized to mean peak height. Peaks included in the further data analysis were those that had good precision in the replicate analyses of the pool. 712 were judged to meet this criterion and used for the further data analysis. These 712 analytes included both unknown and known compounds, comprising metabolites of tyrosine, tryptophan and purine pathways, and some redox active markers of oxidative stress, antioxidants and vitamins [26], [29].

### Data analysis

All chromatograms in the study were processed as previously described [14]. The levels of all analytes were normalized to the averaged pool and expressed as the percentage of those analytes in the pool. Conventional statistical methods and projection to latent structures- discriminant analysis (PLS-DA) [30], were used for the data analysis. Both unprocessed and preprocessed datasets were used for PLS-DA. To avoid possible statistical artifacts, and to find the analytes (potential biomarkers) which can be used to build PLS-DA models for class discrimination and class membership prediction, we used a preprocessing approach. For this, Student's t-tests followed by the area under the Receiver Operating Characteristic (ROC) curve were used. The ROC curve is a plot of the sensitivity (or true-positive rate) to the false-positive rate [31]. Using this approach, values of the analytes in each subgroup were compared to another, e.g., controls vs. *LRRK2* PD patients. The criteria for inclusion of analytes into further analysis were set at: *p* value less than 0.01 and area under the ROC curve larger than 0.8.

To build and validate PLS-DA models for class discrimination and class membership prediction, data for the subjects from different subgroups were randomly divided into the training (∼2/3 of all subjects in a given subgroup) and test (∼1/3 of the subjects) sets. Test sets were excluded from the data pre-processing and model construction. Following construction of PLS-DA models using training sets, the models were then used to predict class membership of the subjects in the test sets. This procedure was repeated four times, different subjects in training and test sets were included and a new PLS-DA model was constructed each time.

## Results

**Figure 1:**
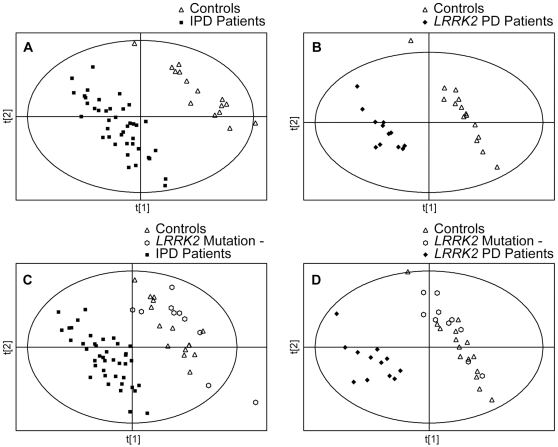
PLS-DA scores plots of control subjects and PD patients. PLS-DA scores plots showing a separation between control subjects (n = 15) and idiopathic Parkinson's Disease (IPD) patients (n = 41), and between control subjects (n = 15) and *LRRK2* PD patients (n = 12). All peaks (no pre-processing) were used for these analyses. The data from control subjects and from the healthy family members of *LRRK2* PD patients without the mutation (n = 10) were used for the analysis either separately (panels A and B), or were combined (panels C and D).

In the initial analysis we analyzed the data from control subjects and idiopathic and *LRRK2* PD patients to determine if there were any differences in the metabolomic profiles between these groups. Since healthy family members of *LRRK2* PD patients without the mutation could be considered as controls, the data from them were also included in this analysis, either separately, or together with control subjects. All peaks detected (unprocessed datasets) were used for the initial analysis. Using PLS-DA we found complete separation between both groups of PD patients and controls. PLS-DA scores plots showing separation between controls and idiopathic PD patients, and between controls and *LRRK2* PD patients, are shown in Figure 1 (panels A and B, respectively). Complete separation was also found when the data from control subjects and from healthy family members of *LRRK2* PD patients without the mutation were combined (Figure 1, panels C and D).

**Figure 2:**
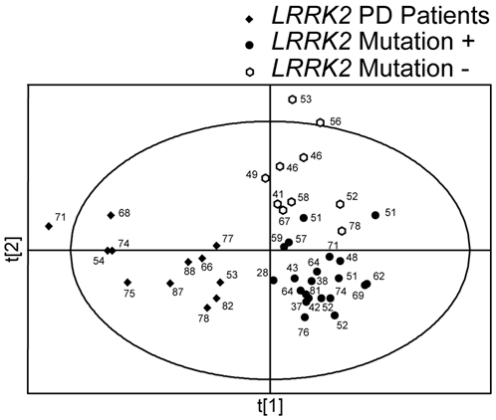
PLS-DA scores plots of *LRRK2* PD patients and their family members. PLS-DA scores plot showing a separation between *LRRK2* PD patients (n = 12) and their healthy family member with (n = 21) or without (n = 10) the gene mutation. All peaks (no pre-processing) were used for this analysis. Ages of the individual subjects are shown next to their symbols.

In order to determine if there were differences in metabolomic profiles associated with the *G2019S* mutation with and without PD, we next analyzed unprocessed data sets from *LRRK2* PD patients and from their family members with and without the mutation. A PLS-DA score plot showing separation between *LRRK2* PD patients and family members is shown in Figure 2. PLS-DA showed complete separation between *LRRK2* patients and their family members; there was, however, some overlap between family members with and without the mutation. *LRRK2* PD patients participating in this study were significantly older than their healthy family members with the mutation (72.8±11.2 vs. 55.2±14.1 years old, respectively, mean±SD, p<0.05). These age differences had no apparent effect on the observed differences in metabolomic profiles, since PLS-DA scores plot didn't show significant separation based on the age of individual subjects (Fig. 2). However, additional studies involving older healthy family members of *LRRK2* PD patients, who carry the mutation, are necessary to validate these findings.

**Figure 3:**
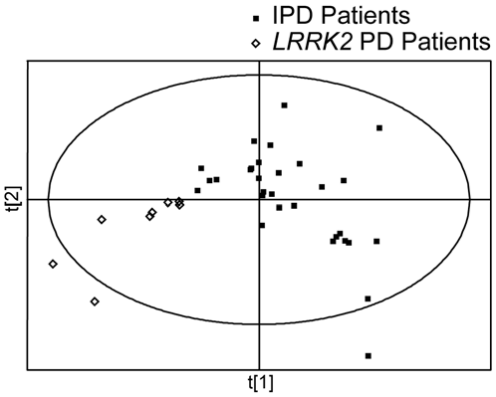
PLS-DA scores plots of IPD and *LRRK2* PD patients. PLS-DA scores plot showing a significant separation between IPD patients (n = 30) and *LRRK2* PD patients (n = 8) using preprocessed datasets (see methods for details). Eleven IPD patients and 4 *LRRK2* PD patients were randomly chosen as the test set and were not used in PLS-DA model construction. Class membership of the subjects in the test set was then predicted using this PLS-DA model shown in Figure 4.

**Figure 4:**
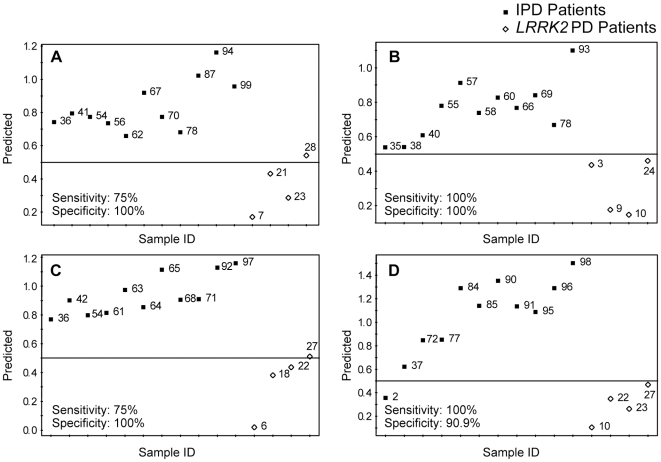
PLS-DA prediction plots of IPD patients and *LRRK2* PD patients. Twelve analytes (Table 3) were used to build PLS-DA separation model, based on randomly selected 30 IPD and 8 *LRRK2* PD patients (a representative plot is shown in Figure 3). The resulting models were used to predict class membership of the remaining 11 IPD and 4 *LRRK2* PD patients. This procedure was carried out 4 times with different IPD and *LRRK2* PD patients included in the test and training sets each time; the results are presented in panels A–D for all four individual models. Predictions were made with a cutoff of 0.5 for class membership. Numbers next to the symbols refer to the sample codes of the subjects.

**Figure 5:**
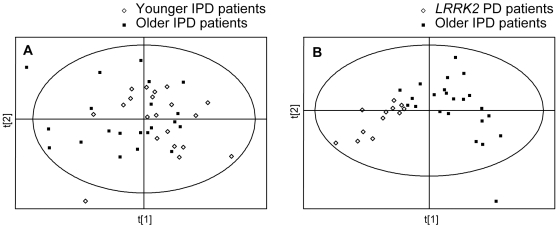
Age effects on metabolomic profiles of IPD patients and *LRRK2* PD patients. (A) PLS-DA scores plot showing lack of separation between younger (57.3±5.6 years old, n = 19, mean±SD) and older (71.4±3.3 years old, n = 22, mean±SD) idiopathic PD patients. (B) PLS-DA scores plot showing a significant separation between older idiopathic PD patients (71.4±3.3 years old, n = 22, mean±SD) and *LRRK2* patients (72.8±11.2 years old, n = 12, mean±SD). The analytes discriminating between all IPD patients and *LRRK2* patients (Figure 4 and Table 3) were used to for the analysis.

In order to find the analytes which could be used to discriminate between idiopathic and *LRRK2* PD patients, we then built PLS-DA models, which were used for class separation and membership prediction, using both unprocessed and pre-processed datasets. The PLS-DA model built using unprocessed datasets showed a complete separation between the two groups. Caution should be exercised to avoid potential overfitting of PLS-DA models for finding separation between different groups. Some concerns related to possible overfitting of PLS-DA models when using datasets, which have the number of variables higher than the number of observations, have been published in recent years [32], [33]. Therefore, we pre-processed our datasets using Student's t-test (*p* value cutoff set at <0.01) followed by the area under the ROC curve (cutoff set at >0.8).Subjects in each group were randomly divided into training and test sets (∼2/3 and ∼1/3 of all subjects in a given subgroup, respectively). This procedure was carried out four times with different subjects included in the test set each time, 12 analytes (Table 3) were used to build the models. A representative PLS-DA separation plot is shown in Figure 3; the individual prediction plots for all four analyses are shown in Figure 4 (panels A to D). Sensitivity and specificity of this approach were found to be 87.5±7.2 and 97.7±2.3 (n = 4, mean±SEM), respectively. There were significant age differences between *LRRK2* and idiopathic PD patients participating in this study; *LRRK2* patients were older than the idiopathic PD patients (72.8±11.2 vs. 64.8±8.4 years old, respectively, mean±SD, p<0.02). Therefore, it is possible that the observed separation between metabolomic profiles of *LRRK2* and idiopathic PD patients may be attributed to the age differences. To address this possibility we separated idiopathic PD patients into older and younger groups, with age of the older group matching the age of *LRRK2* patients (71.4±3.3 and 72.8±11.2 years old, respectively, mean±SD). No separation between the older and the younger groups of idiopathic PD patients was found (Figure 5A). We also analyzed the data for *LRRK2* patients and older idiopathic PD patients separately (the younger group of idiopathic PD patients was excluded from the analysis) and found complete separation between these two groups (Figure 5B). These results indicate that differences in metabolomes of *LRRK2* and idiopathic PD patients are not affected by age.

**Table 3:** Analytes discriminating between idiopathic and *LRRK2* PD.

Retention time	Dominant channel	Change	% of change
19.7	16	−	45
25.9	6	−	87
42.3	13	−	61
67.1	15	−	27
67.4	16	−	19
80.1	4	+	10
91.1	12	−	51
91.7	12	−	21
91.7	16	−	39
93.2	16	+	47
94.2	16	−	42
100.2	10	+	11

Analytes are defined by their retention time and dominant channel in LCECA profiles. + up-regulated in *LRRK2* PD; − down-regulated in *LRRK2* PD.

**Figure 6:**
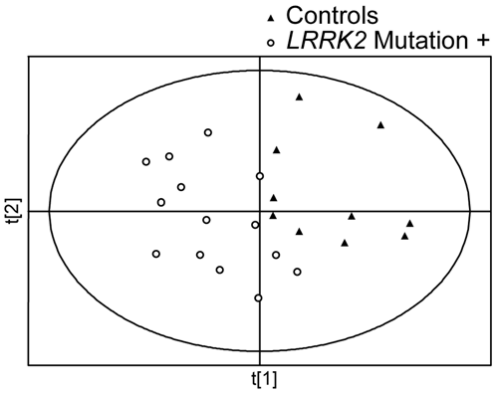
PLS-DA score plots of controls and subjects with *G2019S LRRK2* gene mutation. PLS-DA scores plot showing a significant separation between controls (n = 10) and asymptomatic subjects with *G2019S LRRK2* gene mutation (n = 14) using preprocessed datasets. Five controls subjects and seven mutation carriers were randomly selected as the test set and were not used in PLS-DA model construction. Class membership of the subjects in the test set was predicted using this PLS-DA model shown in Figure 7.

**Figure 7:**
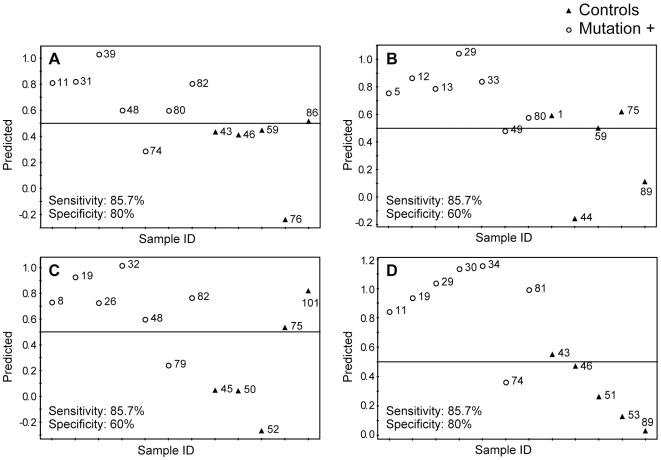
PLS-DA prediction plots of controls and subjects with *G2019S LRRK2* gene mutation. Nine analytes (Table 4) were used to build PLS-DA separation model, based on randomly selected 10 controls and 14 *LRRK2* gene carriers (a representative plot is shown in Figure 6). The resulting models were used to predict class membership of the remaining 5 controls and 7 *LRRK2* gene carriers. This procedure was carried out 4 times with different controls and gene carriers included in the test and training sets each time; the results are presented in panels A–D for all four individual models. Predictions were made with a cutoff of 0.5 for class membership. Numbers next to the symbols refer to the sample codes of the subjects.

We also analyzed the data from control subjects and from asymptomatic *LRRK2* mutation carriers to determine if there are specific metabolomic signatures associated with the mutation. Similar to the analysis of idiopathic and *LRRK2* PD patients, we used preprocessed datasets to build PLS-DA models for class separation and membership prediction. Subjects in each group were randomly divided into training and test sets. This procedure was repeated four times with different subjects used each time, with 9 analytes (Table 4) used for model building. Interestingly, two of the analytes (80.1-4 and 42.3-13) were found in the groups of peaks discriminating between idiopathic and *LRRK2* PD patients and between asymptomatic *LRRK2* mutation carriers and controls. A representative PLS-DA separation plot is shown in Figure 6; the prediction plots for asymptomatic mutation carriers and controls are shown in Figure 7 (panels A to D). Sensitivity and specificity of this approach were found to be 85.7±1.5 and 70.0±5.8 (n = 4, mean±SEM), respectively.

**Table 4:** Analytes discriminating between *LRRK2* mutation carriers and Controls.

Retention time	Dominant channel	Change	% of change
5.0	6	−	52
5.1	12	−	22
15.1	16	−	50
20.7	14	−	36
30.6	14	−	40
42.3	13	−	39
59.4	12	−	43
80.1	4	+	5
82.7	16	−	77

Analytes are defined by their retention time and dominant channel in LCECA profiles.

+ up-regulated in *LRRK2* mutation carriers; − down-regulated in *LRRK2* mutation carriers.

Although the majority of the analytes driving categorical separations between the groups were not known, there were differences in the levels of some known compounds. Similar to our previous report, uric acid (UA) levels were lower in PD patients (both idiopathic and *LRRK2)* compared to controls (18% decrease, p<0.01). There was a slight non-significant ∼10% decrease in UA levels in asymptomatic mutation carriers, compared to controls; more samples from the asymptomatic mutation carriers should be analyzed to determine whether UA levels are significantly decreased in these subjects. Hypoxanthine levels were significantly decreased in the asymptomatic mutation carriers by 25% (*p<*0.05), but not in the idiopathic or *LRRK2* PD patients, when compared to control subjects. We also found that xanthine levels were significantly decreased by 18% (*p*<0.05) in *LRRK2* PD patients compared to controls. Since all PD patients participating in this study were on antiparkinsonian medications, it is possible that in these PD patients, levels of purine metabolites could be affected by the antiparkinsonian treatments. To address this possibility, we reanalyzed part of the samples from our previous study [14], which involved unmedicated PD patients, and found a significant decrease in hypoxanthine levels in unmedicated PD patients, compared to control subjects (45% decrease, *p<*0.05; n = 15 and n = 20, respectively). Levels and ratios of some other purine metabolites were also significantly decreased in unmedicated PD patients (Table 5). Analysis of the data from the patients on different antiparkinsonian medications showed normalization of plasma hypoxanthine levels with levodopa and with combination of levodopa with dopamine receptor agonists.

**Table 5:** Purine metabolites in PD.

	Controls (n = 25)	Unmed PD (n = 15)	% change *(p* value)
HX	177±38	103±21	−41.91 (0.046)
X	96±6	82±6	−14.15 (0.054)
XAN	79±6	105±20	32.16 (0.123)
UA	97±4	83±5	−14.31 (0.050)
G	91±5	84±5	−8.08 (0.164)
HX/XAN	200±35	116±27	−41.83 (0.033)
X/XAN	136±12	101±12	−26.04 (0.021)
HX/UA	183±38	124±21	−32.32 (0.041)

Data are represented as mean±SEM of percentage of plasma pool value. Control group includes both normal control subjects and healthy family members from *LRRK2* PD patients who did not have G2019S mutation. HX–hypoxanthine, X–xanthosine, XAN–xanthine, UA–uric acid, G- guanosine.

## Discussion

The role of genetic factors in the pathogenesis of PD has been proven during the last several years, and several mutations have been implicated in familial PD [1]–[4]. Although these may account for a relatively small percentage of all PD cases, analysis of the pathogenetic mechanisms involving these mutations may provide insights into the pathogenesis of idiopathic PD, it is also possible that different mutations could cause PD via different mechanisms. The autosomal dominant 6055G>A mutation in the *LRRK2* gene, which causes a *G2019S* substitution, is one of the most frequent mutations in familial PD [34]. *LRRK2* PD patients are indistinguishable from idiopathic PD, both clinically and in their response to medication [8], [23], [24]. In central Norway about 3% of PD cases have been found to carry the *G2019S* substitution [8]. In this study we used metabolomic profiling to determine if there are specific metabolomic signatures associated with *LRRK2* PD, compared to idiopathic PD. We also addressed the question whether the asymptomatic *G2019S* mutation carriers could be distinguished by metabolomic analysis from control subjects.

Similar to our previous study [14], we found that metabolomic profiles of idiopathic PD are different from those of age matched control subjects. In this study we demonstrated that PD patients with *LRRK2* mutations are also different from the control subjects. The separation between PD patients and controls raised the question of whether PD medications caused the distinction. Both idiopathic and *LRRK2* PD subjects involved in this study were taking antiparkinsonian medications, and no samples from the unmedicated patients were available. Therefore, it is possible that the observed separation could be related to drug effects, which could involve unknown drug metabolites and drug-induced changes in metabolism. However, we previously demonstrated that metabolomic profiles can differentiate between the medicated PD patients and controls, and this separation is not attributable to drug effects [14]. Additional studies involving a larger population of both medicated and unmedicated *LRRK2* PD patients are necessary to address these issues.

We also found that the genetically determined groups could be separated from the non-genetic groups. There was a clear separation between metabolomic profiles of idiopathic and *LRRK2* PD patients, and this separation was not attributed to the age differences. Based on the limited number of analytes from plasma we were able to predict whether the patients were from idiopathic PD or from *LRRK2* PD groups. PLS-DA models for prediction of the class membership (*LRRK2* PD vs. idiopathic PD) were built four times with different subjects from the groups randomly selected for training and prediction sets each time. Although the number of subjects involved in the study was small, the PLS-DA models were able to accurately predict the class membership. Nevertheless, additional experiments with a larger study size are necessary to confirm these findings.

Analysis of metabolomic profiles of the subjects harboring *LRRK2 G2019S* showed that there were differences dependent on the clinical phenotype: *LRRK2* PD patients were partially separated from their healthy family members carrying the mutation. The observation that there is some overlap between the subjects carrying the mutation with *LRRK2* PD patients suggests the possibility of developing predictive biomarkers for this group, i.e., who amongst the mutation carriers will develop PD in the future. Longitudinal studies should provide more evidence for this possibility.

The majority of the analytes driving separations between the groups were not known. However, the results on known compounds strongly suggest that abnormalities in the purine pathway may be implicated in PD. Similar to the previous studies [14], [35], [36], UA levels were found to be decreased in plasma of PD patients. Several studies provide evidence that UA is involved in the development and progression of PD. Prospective epidemiological studies showed that healthy individuals with higher blood UA levels are at reduced risk for developing PD [37]. A lower risk of PD has been reported among individuals consuming diets that increase serum UA [38]. Higher blood levels of UA in patients recently diagnosed with PD predict a slower rate of disease progression, assessed by both clinical and neuroimaging measures [39]. The mechanisms leading to decreased UA in PD are not known. UA is a major antioxidant, and in humans is the final product of purine metabolism. It is possible that aberrations in the purine pathway in PD could occur upstream from UA. The results of this study support this hypothesis. We found for the first time significant changes in major metabolites of the purine pathway in plasma of PD patients. Notably, hypoxanthine levels were significantly decreased in unmedicated PD patients, as well as in asymptomatic *LRRK2* mutation carriers, compared to controls. Xanthine levels were lower in *LRRK2* PD patients (but not in IPD patients) as compared to controls. These findings suggest a potential role of the purine pathway in the pathogenesis of PD.

In this study LCECA was used for metabolomic profiling. We are currently working on the structural elucidation of unknown biomarkers using different mass spectrometry (MS) approaches [40]. Identification of the individual analytes (potential biomarkers) is crucial since they may play a central role in pathogenesis of the disease. The *G2019S* substitution in *LRRK2* may lead to biochemical changes that are common to PD pathophysiology irrespective of etiology.

An important aspect of this study was to distinguish asymptomatic *G2019S* carriers, representing a group at risk to develop PD, from controls. Obviously, a genetic test provides complete separation of the mutation carriers from non-carriers. The results of this study do not provide direct evidence for the subsets among the *G2019S* mutation carriers. Whether metabolomics approach could be used to predict development of PD in a subset of the *G2019S* carriers remains to be determined. Future longitudinal studies are needed to determine whether one can predict disease penetrance in these subjects. The ability to recognize this group prior to onset of disease indicates the possibility of developing an early diagnostic tool using metabolomics. One could then screen individuals with increased risk of PD including first degree relatives, patients with hyposmia and patients carrying genetic risk factors. Metabolomic profiles could be screened to warn of impending PD symptom onset, and thus allow earlier institution of neuroprotective therapy in presymptomatic patients to slow or prevent nigral cell loss before PD symptomatology becomes evident. Although the present results are promising, this study needs to be repeated in a larger population due to our limitation of a low numbers of cases.

## References

[1] Hardy J, Cai H, Cookson MR, Gwinn-Hardy K, Singleton A (2006). Genetics of Parkinson's disease and parkinsonism.. Ann Neurol.

[2] Klein C, Schlossmacher MG (2007). Parkinson disease, 10 years after its genetic revolution: multiple clues to a complex disorder.. Neurology.

[3] Schiesling C, Kieper N, Seidel K, Kruger R (2008). Review: Familial Parkinson's disease–genetics, clinical phenotype and neuropathology in relation to the common sporadic form of the disease.. Neuropathol Appl Neurobiol.

[4] Thomas B, Beal MF (2007). Parkinson's disease.. Hum Mol Genet.

[5] Bras JM, Guerreiro RJ, Ribeiro MH, Januario C, Morgadinho A (2005). G2019S dardarin substitution is a common cause of Parkinson's disease in a Portuguese cohort.. Mov Disord.

[6] Lesage S, Ibanez P, Lohmann E, Pollak P, Tison F (2005). G2019S LRRK2 mutation in French and North African families with Parkinson's disease.. Ann Neurol.

[7] Ozelius LJ, Senthil G, Saunders-Pullman R, Ohmann E, Deligtisch A (2006). LRRK2 G2019S as a cause of Parkinson's disease in Ashkenazi Jews.. N Engl J Med.

[8] Aasly JO, Toft M, Fernandez-Mata I, Kachergus J, Hulihan M (2005). Clinical features of LRRK2-associated Parkinson's disease in central Norway.. Ann Neurol.

[9] Brucke T, Djamshidian S, Bencsits G, Pirker W, Asenbaum S (2000). SPECT and PET imaging of the dopaminergic system in Parkinson's disease.. J Neurol.

[10] Kim YJ, Ichise M, Ballinger JR, Vines D, Erami SS (2002). Combination of dopamine transporter and D2 receptor SPECT in the diagnostic evaluation of PD, MSA, and PSP.. Mov Disord.

[11] LeWitt PA, Galloway MP, Matson W, Milbury P, McDermott M (1992). Markers of dopamine metabolism in Parkinson's disease. The Parkinson Study Group.. Neurology.

[12] El-Agnaf OM, Salem SA, Paleologou KE, Curran MD, Gibson MJ (2006). Detection of oligomeric forms of alpha-synuclein protein in human plasma as a potential biomarker for Parkinson's disease.. Faseb J.

[13] Tokuda T, Salem SA, Allsop D, Mizuno T, Nakagawa M (2006). Decreased alpha-synuclein in cerebrospinal fluid of aged individuals and subjects with Parkinson's disease.. Biochem Biophys Res Commun.

[14] Bogdanov M, Matson WR, Wang L, Matson T, Saunders-Pullman R (2008). Metabolomic profiling to develop blood biomarkers for Parkinson's disease.. Brain.

[15] Scherzer CR, Eklund AC, Morse LJ, Liao Z, Locascio JJ (2007). Molecular markers of early Parkinson's disease based on gene expression in blood.. Proc Natl Acad Sci U S A.

[16] Goodacre R, Vaidyanathan S, Dunn WB, Harrigan GG, Kell DB (2004). Metabolomics by numbers: acquiring and understanding global metabolite data.. Trends Biotechnol.

[17] Brindle JT, Antti H, Holmes E, Tranter G, Nicholson JK (2002). Rapid and noninvasive diagnosis of the presence and severity of coronary heart disease using 1H-NMR-based metabonomics.. Nat Med.

[18] Griffin JL, Nicholls AW (2006). Metabolomics as a functional genomic tool for understanding lipid dysfunction in diabetes, obesity and related disorders.. Pharmacogenomics.

[19] Di Leo A, Claudino W, Colangiuli D, Bessi S, Pestrin M (2007). New strategies to identify molecular markers predicting chemotherapy activity and toxicity in breast cancer.. Ann Oncol.

[20] Holmes E, Tsang TM, Huang JT, Leweke FM, Koethe D (2006). Metabolic profiling of CSF: evidence that early intervention may impact on disease progression and outcome in schizophrenia.. PLoS Med.

[21] Rozen S, Cudkowicz ME, Bogdanov M, Matson WR, Kristal BS (2005). Metabolomic analysis and signatures in motor neuron disease.. Metabolomics.

[22] Underwood BR, Broadhurst D, Dunn WB, Ellis DI, Michell AW (2006). Huntington disease patients and transgenic mice have similar pro-catabolic serum metabolite profiles.. Brain.

[23] Goldwurm S, Zini M, Di Fonzo A, De Gaspari D, Siri C (2006). LRRK2 G2019S mutation and Parkinson's disease: a clinical, neuropsychological and neuropsychiatric study in a large Italian sample.. Parkinsonism Relat Disord.

[24] Khan NL, Jain S, Lynch JM, Pavese N, Abou-Sleiman P (2005). Mutations in the gene LRRK2 encoding dardarin (PARK8) cause familial Parkinson's disease: clinical, pathological, olfactory and functional imaging and genetic data.. Brain.

[25] Gelb DJ, Oliver E, Gilman S (1999). Diagnostic criteria for Parkinson disease.. Arch Neurol.

[26] Kristal BS, Vigneau-Callahan KE, Matson WR (1998). Simultaneous analysis of the majority of low-molecular-weight, redox-active compounds from mitochondria.. Anal Biochem.

[27] Shi H, Vigneau-Callahan KE, Matson WR, Kristal BS (2002). Attention to relative response across sequential electrodes improves quantitation of coulometric array.. Anal Biochem.

[28] Vigneau-Callahan KE, Shestopalov AI, Milbury PE, Matson WR, Kristal BS (2001). Characterization of diet-dependent metabolic serotypes: analytical and biological variability issues in rats.. J Nutr.

[29] Kristal BS, Vigneau-Callahan KE, Moskowitz AJ, Matson WR (1999). Purine catabolism: links to mitochondrial respiration and antioxidant defenses?. Arch Biochem Biophys.

[30] Eriksson L, Johansson E, Kettanah-Wold N, Wold S (2001). Multi- and megavariate data analysis: Sweden: Umetrics AB, Malmo..

[31] Broadhurst DI, Kell DB (2006). Statistical strategies for avoiding false discoveries in metabolomics and related experiments.. Metabolomics.

[32] Rubingh CM, Bijlsma S, Derks EPPA, Bobeldijk I, Verheij ER (2006). Assessing the performance of statistical validaiton tools megavariate metabolomics data.. Metabolomics.

[33] Westerhuis JA, Hoefsloot HCJ, Smit S, Vis DJ, Smilde AK (2008). Assessment of PLSDA cross validation.. Metabolomics.

[34] Healy DG, Falchi M, O'Sullivan SS, Bonifati V, Durr A (2008). Phenotype, genotype, and worldwide genetic penetrance of LRRK2-associated Parkinson's disease: a case-control study.. Lancet Neurol.

[35] Annanmaki T, Muuronen A, Murros K (2007). Low plasma uric acid level in Parkinson's disease.. Mov Disord.

[36] de Lau LM, Koudstaal PJ, Hofman A, Breteler MM (2005). Serum uric acid levels and the risk of Parkinson disease.. Ann Neurol.

[37] Ascherio A, LeWitt PA, Xu K, Eberly S, Watts A (2009). Urate predicts rate of clinical decline in Parkinson disease.. Arch Neurol.

[38] Gao X, Chen H, Choi HK, Curhan G, Schwarzschild MA (2008). Diet, urate, and Parkinson's disease risk in men.. Am J Epidemiol.

[39] Schwarzschild MA, Schwid SR, Marek K, Watts A, Lang AE (2008). Serum urate as a predictor of clinical and radiographic progression in Parkinson disease.. Arch Neurol.

[40] Gamache PH, Meyer DF, Granger MC, Acworth IN (2004). Metabolomic applications of electrochemistry/mass spectrometry.. J Am Soc Mass Spectrom.

